# Supplementary material for: Highly defective ultra-small tetravalent MOF nanocrystals
Source: Nat Commun. 2024 Apr 23;15:3434. doi: 10.1038/s41467-024-47426-x (PMC11039632; doi:10.1038/s41467-024-47426-x)
Supplement: Supplementary file 1 — Supplementary Information [file 41467_2024_47426_MOESM1_ESM.pdf]

Supplementary Information

Highly defective ultra-small tetravalent MOF nanocrystals

Shan Dai,[1,4] Charlotte Simms,[2] Gilles Patriarche,[3] Marco Daturi,[4] Antoine Tissot,[1] Tatjana N. Parac-Vogt,[2*] Christian Serre[1*]*

[1] *Institut des Matériaux Poreux de Paris, Ecole Normale Supérieure, ESPCI Paris, CNRS, PSL University, 75005 Paris, France; Email: antoine.tissot@ens.psl.eu; christian.serre@ens.psl.eu*

[2] *Laboratory of Bioinorganic Chemistry, Department of Chemistry, KU Leuven, Celestijnenlaan 200F, 3001 Leuven, Belgium; Email: tatjana.vogt@kuleuven.be*

[3] *Université Paris-Saclay, CNRS, Centre de Nanosciences et de Nanotechnologies, 91120 Palaiseau, France*

[4] *Normandie Univ., ENSICAEN, UNICAEN, CNRS, Laboratoire Catalyse et Spectrochimie, 14000 Caen, France*

Supplementary Methods

All chemicals were purchased from commercial suppliers (Fisher, Acros, Sigma, and TCI), and used as received without further purification.

Room temperature Powder X-ray Diffraction (PXRD) data were recorded on a high-throughput Bruker D8 Advance diffractometer working on transmission mode and equipped with a focusing Göbel mirror producing CuKα radiation (λ = 1.5418 Å) and a LynxEye detector. Temperature dependent PXRD data were recorded with sample closely packed in quartz capillary on a PANalytical EMPYREAN diffractometer with CuKα radiation (λ = 1.5418 Å) and equipped with a HTK-1200N (Anton Parr) high-temperature chamber and a GaliPIX^{3D} detector. ^{1}H NMR spectra were recorded on a Bruker Avance 300 or 400 spectrometer. Thermo Fisher MegaFuge 16R Benchtop was used to isolate the MOF nanoparticles from suspension. Nitrogen porosimetry data were collected on a Micromeritics Tristar/ Triflex instrument at 77 K (pre-activating samples at 70 °C under vacuum, 10 hours). Scanning Electron Microscopy coupled with energy-dispersive X-ray spectroscopy (SEM-EDX) results were recorded with FEI Magellan 400 scanning electron microscope. Thermogravimetric analysis (TGA) data were collected on Mettler Toledo TGA/DSC 2, STAR System apparatus with a heating rate of 5 °C/min under the oxygen flow (40 mL/min). To be noted, for the sake of comparison, the TGA data in main text is normalized with the residual ZrO_2 at 600 °C. Routine infrared spectra were measured with a Nicolet iS5 FTIR ThermoFisher spectrometer. Zeta potential and DLS size measurements of hydrodynamic radii were made on a Malvern Zetasizer Nano-ZS (Malvern Instruments). High resolution TEM images (HRTEM) were acquired on a Titan Themis 200 microscope operating at 200 kV. This microscope was equipped with a Ceta 16M hybrid camera from ThermoFischer Scientific capable of working under low electron irradiation conditions. The HRTEM images were obtained in low dose condition with an irradiation current between 100 and 250 electrons per square angstroms. For the TEM grid preparation, a 2 µl drop of the solution was placed on a 200 mesh copper grid covered with a pure carbon membrane (from Ted Pella).

General synthesis method for HD-US-UiO-66-X

In a typical synthesis, M(IV)$_6$ oxoclusters (0.06 mmol, 75 mg) (following the previously reported protocol[1]) were dispersed in acetic acid (0.5 mL) under stirring at 600 rpm. H_2O (1.25 mL) was subsequently added, and the reaction mixture was stirred until it became completely clear. Ethanol was introduced into the solution followed by the immediate addition of 1.2 mmol BDC-X, and the suspension was stirred for 2h at room temperature. Note, with the reaction proceeds, the suspended linkers were fully dissolved. The resulting solution was evaporated by rotary evaporation at room temperature until approximately 10 mL volume was left. The colloidal suspension was centrifuged at 14,500 rpm for 60 min and then washed twice with the mixture of 20 mL of acetone and 20 mL of ethanol (14,500 rpm, 1.5h). The collected solid was dried under vacuum for 3 h for characterizations and applications. The particle size can be controlled simply by using different amounts of ethanol in the synthesis batch.

Note: the yield of HD-US-UiO-66-X synthesis is highly dependent on the isolation process, particularly of the centrifugation conditions, as it is extremely difficult to fully recover these colloidal stable MOF nanoparticles from the solution. With the above-described process, the yield is around 50-60%. However, this can be improved to 75% by using an ultrahigh-speed centrifuge (18,000 rpm). Depending on the instrument accessibility, we strongly suggest using centrifuge speed as high as possible to obtain the highest product yield.

Synthesis of HD-US-UiO-66-Br (4 nm)

Zr_6 oxoclusters (0.06 mmol, 75 mg) were dispersed in acetic acid (0.5 mL, 8.75 mmol) under stirring at 600 rpm. H_2O (1.25 mL) was subsequently added, and the reaction mixture was stirred until it became completely clear. 20 mL of ethanol was introduced into the solution followed by the immediate addition

of 2-bromo-1,4-dicarboxylic acid (BDC-Br, 72 mg, 0.3 mmol), and the reaction was stirred for 2h at room temperature. The resulting solution was evaporated by rotary evaporation at room temperature until approximately 5 mL volume was left. The colloidal suspension was centrifuged at 14,500 rpm for 60 min and then washed twice with the mixture of 20 mL of acetone and 20 mL of ethanol (14,500 rpm, 1.5h). The collected solid was dried under vacuum for 3 h for characterizations and applications.

Synthesis of HD-US-UiO-66-NO_2 (5 nm)

Zr_6 oxoclusters (0.06 mmol, 75 mg) were dispersed in acetic acid (0.5 mL, 8.75 mmol) under stirring at 600 rpm. H_2O (1.25 mL) was subsequently added, and the reaction mixture was stirred until it became completely clear. 20 mL of ethanol was introduced into the solution followed by the immediate addition of 2-nitrobenzene-1,4-dicarboxylic acid (BDC-NO_2, 62 mg, 0.3 mmol), and the reaction was stirred for 2h at room temperature. The resulting solution was evaporated by rotary evaporation at room temperature until approximately 5 mL volume was left. The colloidal suspension was centrifuged at 14,500 rpm for 60 min and then washed twice with the mixture of 20 mL of acetone and 20 mL of ethanol (14,500 rpm, 1.5h). The collected solid was dried under vacuum for 3 h for characterizations and applications.

Synthesis of HD-US-UiO-66-$(OH)_2$ (4 nm)

Zr_6 oxoclusters (0.06 mmol, 75 mg) were dispersed in acetic acid (0.5 mL, 8.75 mmol) under stirring at 600 rpm. H_2O (1.25 mL) was subsequently added, and the reaction mixture was stirred until it became completely clear. 15 mL of ethanol was introduced into the solution followed by the immediate addition of 2,5-dihydroxy-1,4-dicarboxylic acid (BDC-$(OH)_2$, 60 mg, 0.3 mmol), and the reaction was stirred for 1h at room temperature. The resulting solution was evaporated by rotary evaporation at room temperature until approximately 2-5 mL volume was left. The colloidal suspension was centrifuged at 14,500 rpm for 45 min and then washed twice with the mixture of 20 mL of acetone and 20 mL of ethanol (14,500 rpm, 1.5h). The collected solid was dried under vacuum for 3 h for characterizations and applications.

Synthesis of HD-US-MOF-801 (4 nm)

Zr_6 oxoclusters (0.06 mmol, 75 mg) were dispersed in acetic acid (0.5 mL, 8.75 mmol) under stirring at 600 rpm. H_2O (1.25 mL) was subsequently added, and the reaction mixture was stirred until it became completely clear. 20 mL of ethanol was introduced into the solution followed by the immediate addition of fumaric acid (35 mg, 0.3 mmol), and the reaction was stirred for 1h at room temperature. The resulting solution was evaporated by rotary evaporation at room temperature until approximately 5 mL volume was left. The colloidal suspension was centrifuged at 14,500 rpm for 45 min and then washed twice with the mixture of 20 mL of acetone and 20 mL of ethanol (14,500 rpm, 1.5h). The collected solid was dried under vacuum for 3 h for characterizations and applications.

Synthesis of HD-US-UiO-66(Hf) (5 nm)

Hf_6 oxoclusters (0.06 mmol, 85 mg) were dispersed in acetic acid (0.5 mL, 8.75 mmol) under stirring at 600 rpm. H_2O (1.25 mL) was subsequently added, and the reaction mixture was stirred until it became completely clear. 80 mL of ethanol was introduced into the solution followed by the immediate addition of 1,4-benzenedicarboxylic acid (BDC, 50 mg, 0.3 mmol), and the reaction was stirred for 2h at room temperature. The resulting solution was evaporated by rotary evaporation at room temperature until approximately 10 mL volume was left. The colloidal suspension was centrifuged at 14,500 rpm for 60 min and then washed twice with the mixture of 20 mL of acetone and 20 mL of ethanol (14,500 rpm, 1.5h). The collected solid was dried under vacuum for 3 h for characterizations and applications. The size control approach here can be applied with different amount of ethanol used in the synthesis batch.

Synthesis of HD-200-UiO-66-NH_2

Zr_6 oxoclusters (0.06 mmol, 75 mg) were dispersed in acetic acid (2.5 mL, 44 mmol) under stirring at 600 rpm. H_2O (1.25 mL) was subsequently added, and the reaction mixture was stirred until it became completely clear. 7.5 mL of ethanol was introduced into the solution followed by the immediate addition of 2-aminobenzene-1,4-dicarboxylic acid (BDC-NH_2, 55 mg, 0.3 mmol), and the reaction was stirred for 3h at room temperature. The resulting solution was centrifuged at 14,500 rpm for 45 min and then washed twice with ethanol (14,500 rpm, 45 min). The collected solid was dried under vacuum for 3 h for characterizations and applications.

FTIR spectroscopy with probe molecules

The samples (22 mg) were pressed into self-supporting discs of 2 cm^2 area. The sample disc was placed in an IR quartz cell equipped with KBr windows. A movable quartz sample holder allowed us to put the sample in the infrared beam for IR measurements or into the furnace for thermal activation. The cell is connected to a vacuum line for evacuation, calcination, and introduction of doses of gases. Spectra were all recorded at room temperature. Prior to CD_3CN adsorption, the compounds were pretreated in vacuum at 373 K for 10 h. Transmission spectra were recorded in the 500−4000 cm^{-1} range at 4 cm^{-1} resolution on a Nicolet Nexus spectrometer equipped with an extended KBr beam splitting device and a mercury cadmium. By subtracting the spectrum of the pure UiO-66 from those of CD_3CN adsorbed onto the supported samples, we collected the spectra shown in the main text.

^{1}H NMR spectroscopy

30 mg of MOF were dissolved with ca. 0.5g KOH in 1 mL D_2O in a centrifuge tube. After shaking the tube, the tube was kept at 60 °C for 16 hours to fully digest the MOF. The mixtures were centrifuged and the supernatant was transferred into an NMR tube for measurement.

Gas adsorption experiments

Each 77K N_2 isotherm was using around 50-100 mg of MOF powder. The MOF powder was first loaded in a glass tube and thermally activated at 70 °C under dynamic vacuum for 10 hours before the measurement.

pH value measurement

The pH value of Zr_6 acetate oxoclusters solutions were measured in 5 mL, 20 mL, and 80 mL of water with 0.3 g Zr_6 acetate oxoclusters, giving values between 3.6 and 4.4.

Glycylglycine Hydrolysis

Prior to hydrolysis, MOFs were activated at 120 °C for 20 h. To a 1 mL glass vial was added 2 µmoles of MOF and 950 µl D_2O. Next, 50 µl of a 40 mM solution of Glycylglycine in D_2O was added, the mixture pD was adjusted to 7.4 and incubated at 60 °C with stirring. Vessel was prepared per time point in triplicate. Reactions were stopped at 1 h, 2 h, 5 h, 20 h, 24 h and 48 h and centrifuged at 14,000 rpm for 10 minutes. 500 µl of supernatant was analyzed with ^{1}H-NMR using 3 µl of 0.1M $TMSP_{d4}$ internal standard. MOFs were washed in acetone and analyzed with PXRD after reaction to check their structure integrity.

For recycling experiments, after reaction, the MOFs were washed in D_2O overnight to remove adsorbed substrate and product, followed by washing in 10 mL of acetone and drying in the oven for 8 h at 110 °C. After which the reaction was repeated.

For reactions at different concentrations of glycylglycine, the reaction was performed as above, but with 200 mM and 500 mM glycylglycine.

The MOF underwent thermal activation at 120 °C for 20 h before being incubated in a solution of GG in D_2O, pD = 7.4, 60 °C for 24h. ^{1}H-NMR was used to determine the amount of Glycylglycine, Glycine (G) and cyclic glycylglycine (cGG) in the reaction supernatant after different time points, and rates

determined using pseudo 1st order kinetics (Figures S30, S31). After reaction, the MOF was analyzed with PXRD, confirming that the MOF remained intact.

We started from the pristine HD-US-UiO-66 as it has the largest pore size among the MOFs synthesized in this work. Interestingly, the HD-US-UiO-66 showed a significantly greater (4.6 times) hydrolysis rate compared to the rate of large-sized (above 200nm) UiO-66 reported by some of us under identical conditions (Figure 5b). [2]

Supplementary Discussion

Modulator-induced defect approach (MIDA)

We followed the conventional MIDA, as well as the synthetic parameters of 40 nm UiO-66 and carried out a set of synthetic experiments by reducing the amount of acetic acid by 50% and 84%, respectively. Noteworthy, this conventional strategy also resulted in the generation of very small nanoparticles denoted as MI-US-UiO-66 (MI: modulator induced, US: ultrasmall) of average size close to 5 nm (HRTEM, Figure S12). The PXRD measurements suggested the correlation between the nanoparticle size and the quantity of modulator (Figure S13) due to the clear peak broadening. However, TGA analysis (Figure S14) revealed a strong dependence of the defect number on the quantity of acetic acid applied in the synthesis. More specifically, MI-US-UiO-66 exhibited the lowest defect content (linker: Zr_6=4.7:1). This value was very close to the initial ratio of the precursors, where linker: Zr_6=4.73:1. When, keeping all other conditions equal, one further tuned the ratio of linker:Zr_6 to 6, in line with the theoretical stoichiometry for non-defective UiO-66, the resulted nanoMOF showed an even higher linker to metal node ratio (4.9 :1), indicating only 1.1 linker per formula was missing (TGA, Figure S15). These results aligned very well with previous findings where the missing linker defect content strongly depended on the modulator quantity. Notably, when adding even more acetic acid no change in the final stoichiometry occurred most likely due to the inhibited deprotonation of acetic acid at higher concentration (Figure S16). The comparison of the pore size distributions (Figure S17) between the 40 nm UiO-66 and MI-US-UiO-66, distinct from HD-US-UiO-66, revealed the slightly reduced pore size of MI-US-UiO-66, further implying that the modulator-induced size reduction leads to less defective UiO-66.

Thermal stability of highly defective ultrasmall UiO-66

It is difficult to monitor the thermal stability of ultrasmall MOF nanoparticles via *in situ* thermal PXRD due to the absence of Bragg diffractions. Thus, to analyse the impact of the downsizing over the thermal stability of HD-US-UiO-66, we prepared slightly larger (~7 nm) nanoparticles through our controllable strategy to carry out a variable temperature PXRD experiments. Noteworthy, the nanomaterial remained crystalline up to 300 ºC (Figure S22), against typically 450°C for the bulk UiO-66(Zr). However, such a stability is acceptable for that small and defective MOF nanoparticles, and hence can be potentially promising for many applications requiring high temperature.

Photoluminescence properties of HD-US-UiO-66-NH_2

Luminescence sensing using MOFs is one widely investigated application that takes the advantages of MOFs, such as high surface area, and tunable properties/selectivity. [11] However, as vast majority of sensing applications are in liquid phase, [11] the light scattering along the optical pathway shall be minimized as much as possible. In some cases, the precipitation of luminescent MOFs in solution can result in irreproducible measurements. Accordingly, it is of critical importance to have porous solids that are optically stable. UiO-66-NH_2 is one benchmark luminescent MOF used in the sensing thanks to its inherent luminescence and high chemical stability. The HD-US-UiO-66-NH_2 synthesized in this work was evaluated as a case study. As shown in Figure S24, the time-dependent fluorescence measurements revealed the emission of HD-US-UiO-66-NH_2 was entirely constant for 6h, manifesting very excellent optical stability. The transparency of the MOF suspension was evidenced by both the low signal-to-noise from the spectra and the photography shown in Figure S24. In a nutshell, the above results evidenced the unprecedented advantage of HD-US-UiO-66-NH_2 nanoparticles in reproducible sensing applications.

Synthesis extension to other functional MOFs.

We explored the synthesis of ultra-small UiO-66-X derivatives (X = NH_2, NO_2, $(OH)_2$, Br). As shown in Figure S23a-d, very similar sizes from 4 to 6 nm were obtained with broad PXRD patterns (Figure

S25), and clear diffraction rings from SAED (Figure S26). To produce these derivatives with similar size, less EtOH was needed compared to the parent UiO-66, especially in the case of UiO-66-$(OH)_2$, due to the better solubility of these functionalized ligands. Remarkably, TGA analysis in Figure S27 (summarized in Table S1) indicated that all nanoMOFs exhibited very similar connectivity (4 linkers per formula) as the pristine UiO-66 except for UiO-66-Br (3.3 linkers per formula). The EDX mapping of UiO-66-Br in Figure S28 revealed a linker to Zr_6 ratio of 3.4, in agreement with TGA result. For this latter, one can tentatively assume that the stronger steric hindrance of Br atoms further promotes the formation of ligand defects. Nitrogen porosimetry of the synthesized derivatives also showed enhanced sorption capacities (see Figures S29, Table 1), confirming the high quality of these HD-US-UiO-66-X. Afterwards, to check if our new method could be extended to other MOFs, we explored the synthesis of the Zr-fumarate/MOF-801 as well as replacing Zr_6 by Hf_6 oxocluster as inorganic node. Interestingly, the change in size, solubility of the linker and/or the metal(IV) species did not affect the formation of ultra-small highly defective nanoMOFs as evidenced by HRTEM (Figure S23e, f), PXRD (Figure S30), and TGA (Figure S31, summarized in Table 1) (denoted as HD-US-UiO-66(Hf) and HD-US-MOF-801). The enlargement in Figure S23f present a nanoparticle built with a single unit cell single crystal. This fits well with excellent solubility of fumaric acid in EtOH, again demonstrating the versatility of our method in producing diverse ultra-small UiO-type frameworks with high defect content.

Supplementary Tables

Supplementary Table 1. Connectivity and porosity information of a series of highly defective ultrasmall MOFs synthesized in this work.

**Materials**	**BET Surface area (m^2/g)**	**Total Pore volume (cm^3/ g)**	**Connectivity* (linker per metal node)**
UiO-66	1040	0.51	3.9
UiO-66-NH_2	878	0.42	4.0
UiO-66-NO_2	428	0.21	4.2
UiO-66-$(OH)_2$	637	0.31	3.9
UiO-66-Br	535	0.28	3.3
UiO-66(Hf)	594	0.28	3.9
MOF-801	595	0.28	4.1

**: Theoretical connectivity of linker per metal node is 6.*

Supplementary Table 2. Summary of reported works that dealt with defect-engineering on UiO-66.

MOF	Missing linker/ %	Size	Surface area (m^2/g)	Reference
UiO-66	27%	7 µm	NA	[3]
UiO-66	16%	300-600 nm	NA	[4]
UiO-66	29%	300 nm	1520	[5]
UiO-66	5%	10 nm	1130/1250	[5]
UiO-66	10%	120 nm	1343	[6]
UiO-66	17%	250 nm	1391	[6]
UiO-66	29%	1000 nm	1479	[6]
UiO-66	16%	200 nm	1546	[7]
UiO-66-NDC	0.3%	100 nm	1272	[8]
UiO-66-NDC	12.6%	740 nm	1319	[8]
UiO-66	5%	20 nm	1376	[9]
UiO-66	4%	20nm	700	[10]
**UiO-66 (HD-US-UiO-66-X)**	**35%**	**4-6 nm**	**428-1040**	**This work**

Supplementary Figures

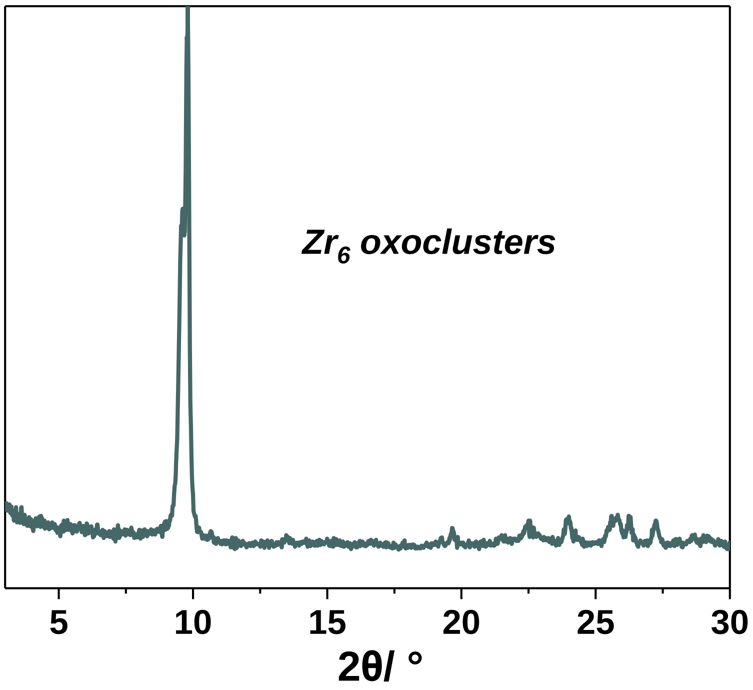

Supplementary Figure 1. PXRD pattern (λ = 1.5418 Å) of the synthesized Zr_6 oxoclusters.

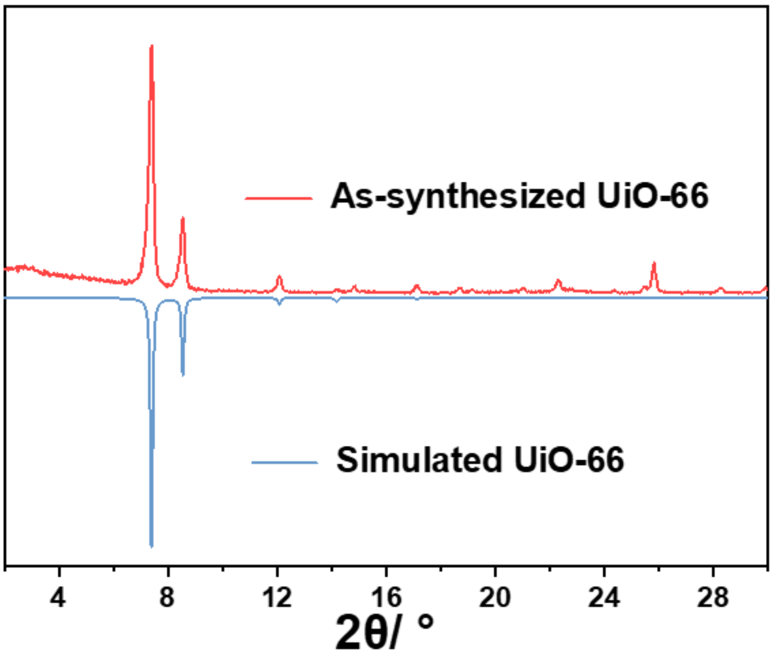

Supplementary Figure 2. PXRD pattern (λ = 1.5418 Å) of the synthesized UiO-66 compared to the simulated UiO-66.

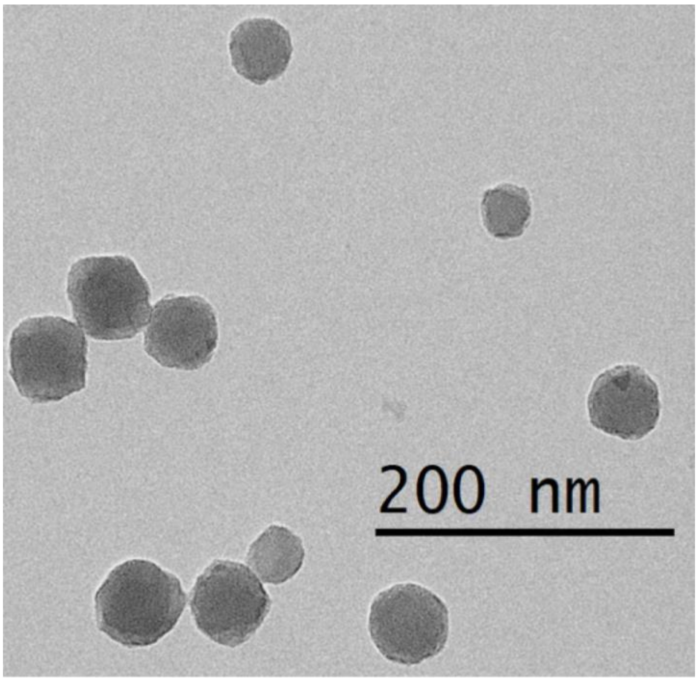

Supplementary Figure 3. TEM image of the synthesized UiO-66 (ca. 40 nm).

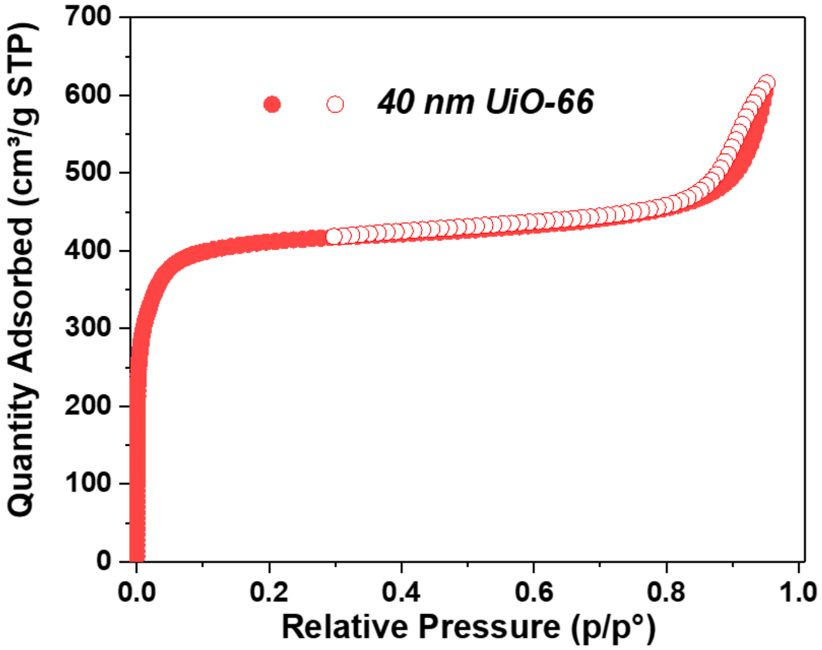

Supplementary Figure 4. 77K N_2 sorption isotherm of 40 nm UiO-66.

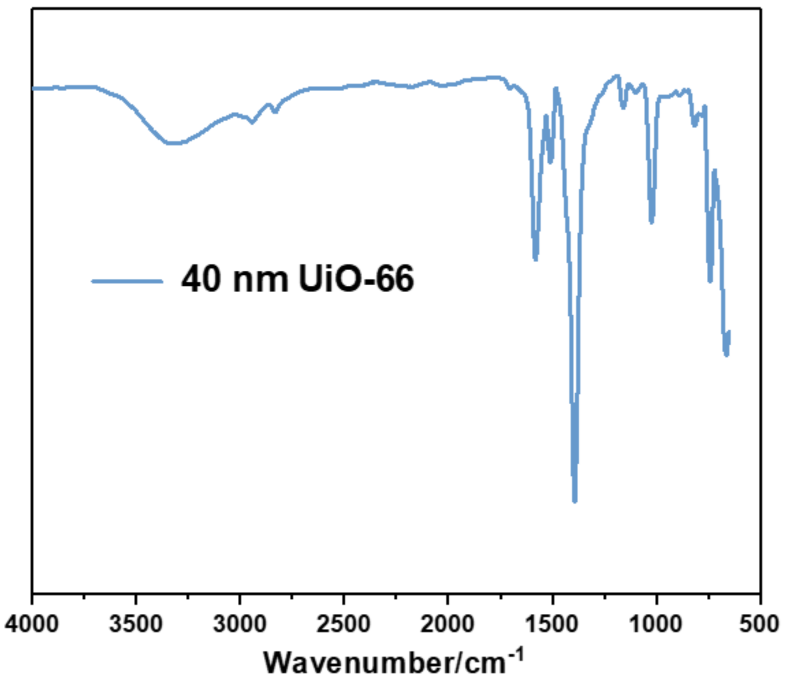

Supplementary Figure 5. FTIR spectrum of 40 nm UiO-66.

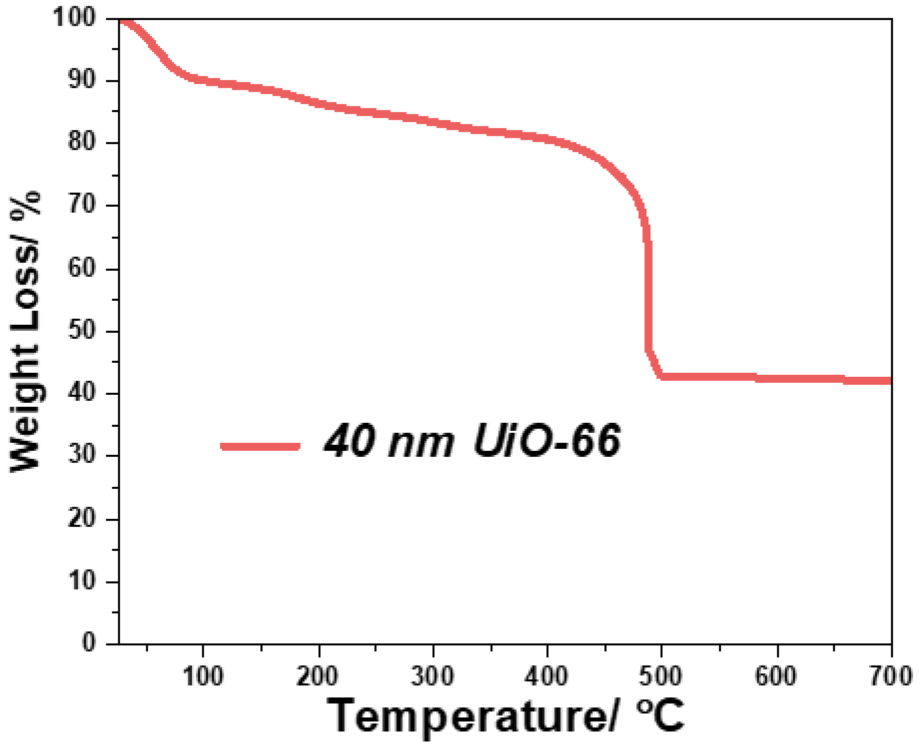

Supplementary Figure 6. TGA of 40 nm UiO-66, ran under oxygen flow, 70 mL/min, 5 °C/min.

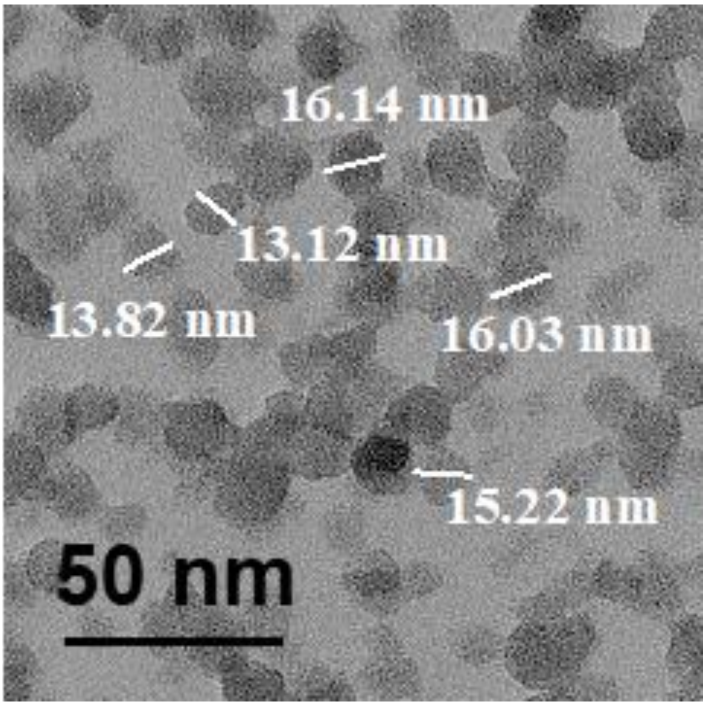

Supplementary Figure 7. TEM image of 14 nm UiO-66 (synthesized with 20 mL EtOH).

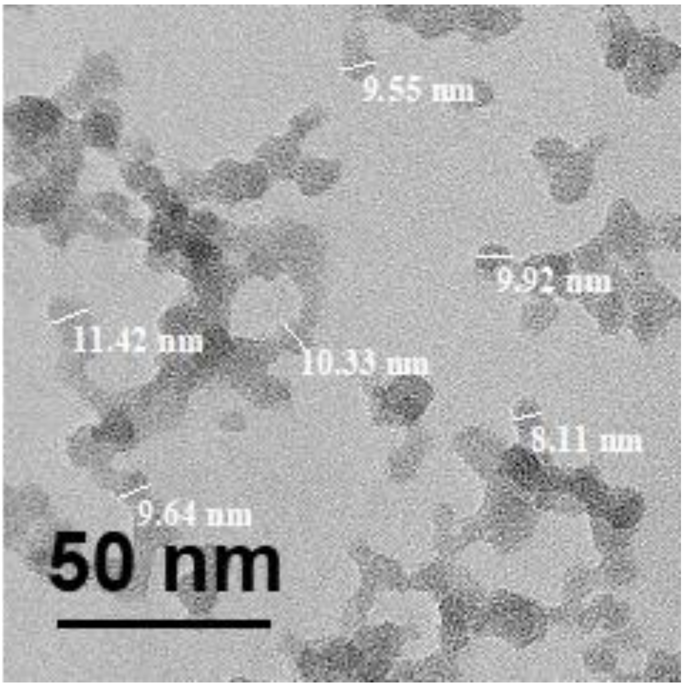

Supplementary Figure 8. TEM image of 9 nm UiO-66 (synthesized with 40 mL EtOH).

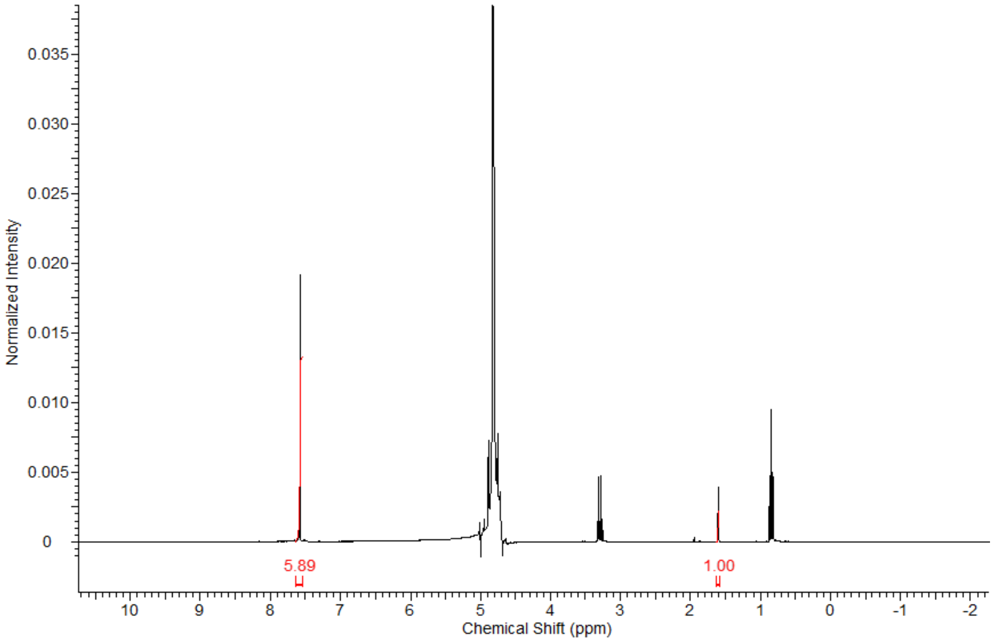

Supplementary Figure 9. ^{1}H-NMR spectra of HD-US-UiO-66.

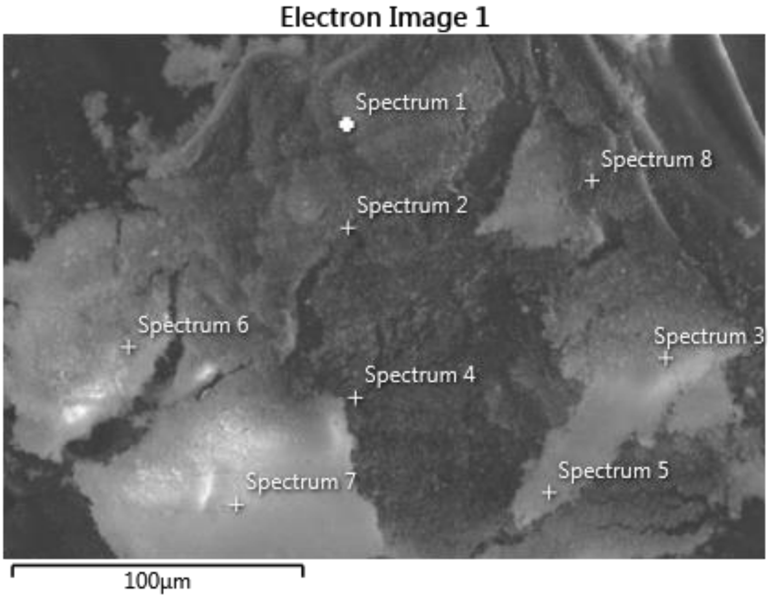

Statistics	Cl (at%)	Zr (at%)
Max	8.02	91.98
Min	6.25	93.75
Average	7.30	92.70

Supplementary Figure 10. EDX analysis of 40 nm UiO-66 and the corresponding normalized atomic ratio of Cl and Zr.

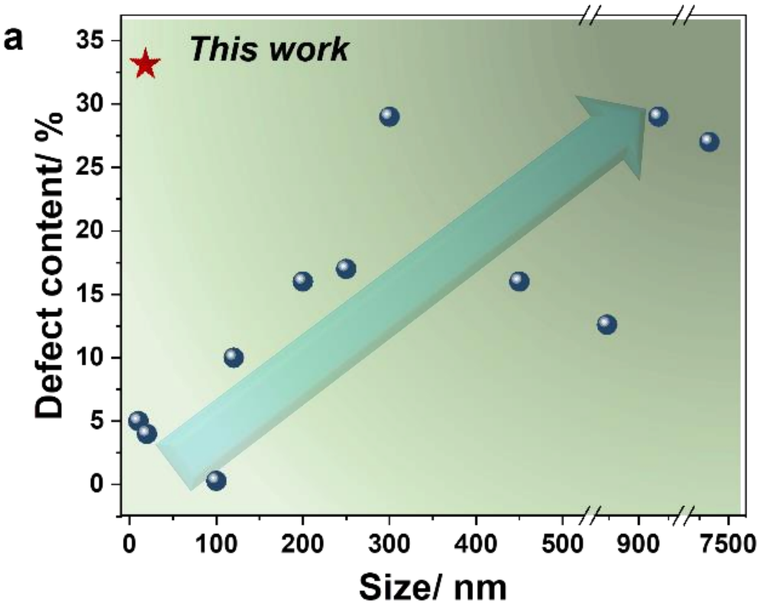

Supplementary Figure 11. Plots of defect content versus the size of MOF of the published articles (adapted from Table S2) and the result from this work.

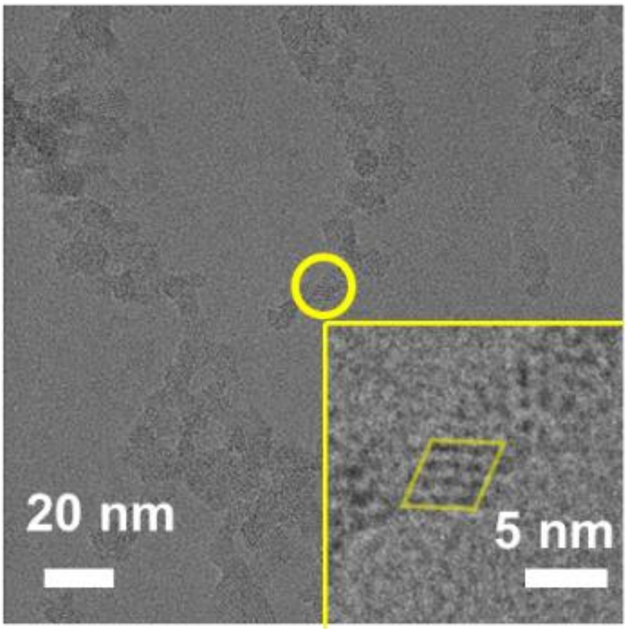

Supplementary Figure 12. HRTEM image of the ultra-small UiO-66 synthesized using 0.08 mL acetic acid.

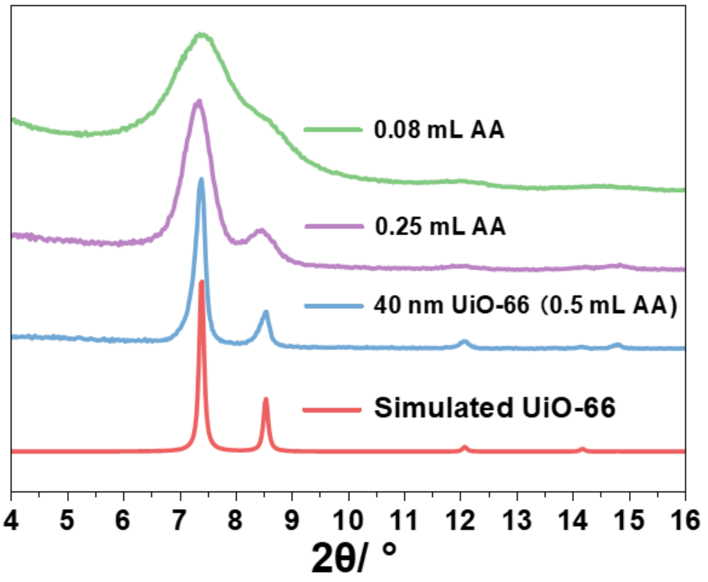

Supplementary Figure 13. PXRD patterns of UiO-66 after the reduction of the quantity of acetic acid (AA) in comparison to the calculated pattern.

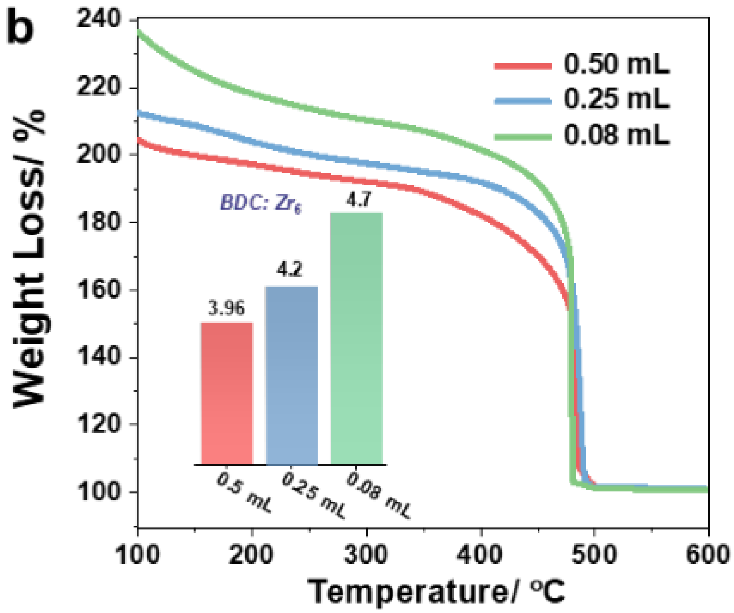

Supplementary Figure 14. TGA results of the UiO-66 samples synthesized with different amounts of acetic acid (0.5, 0.25, 0.08 mL), ran under oxygen flow, 70 mL/min, 5 °C/min.

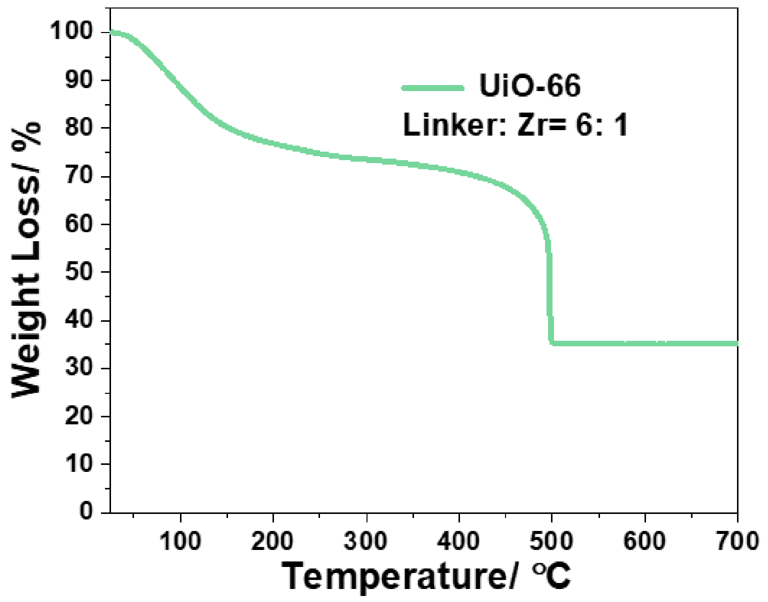

Supplementary Figure 15. TGA of MI-US-UiO-66 synthesized with more linker (Linker:Zr_6=6:1), ran under oxygen flow, 70 mL/min, 5 °C/min.

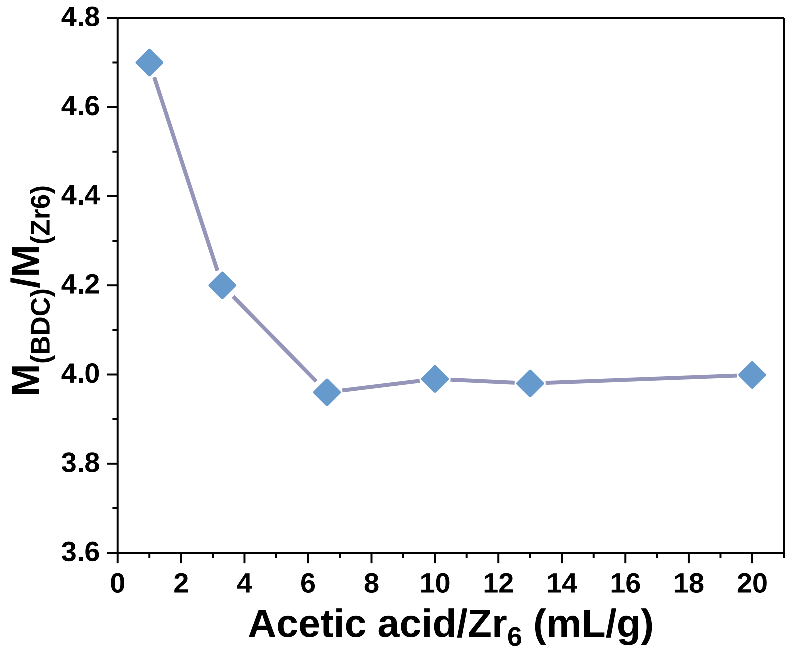

Supplementary Figure 16. Defect content of different UiO-66 particles synthesized using different amount of acetic acid.

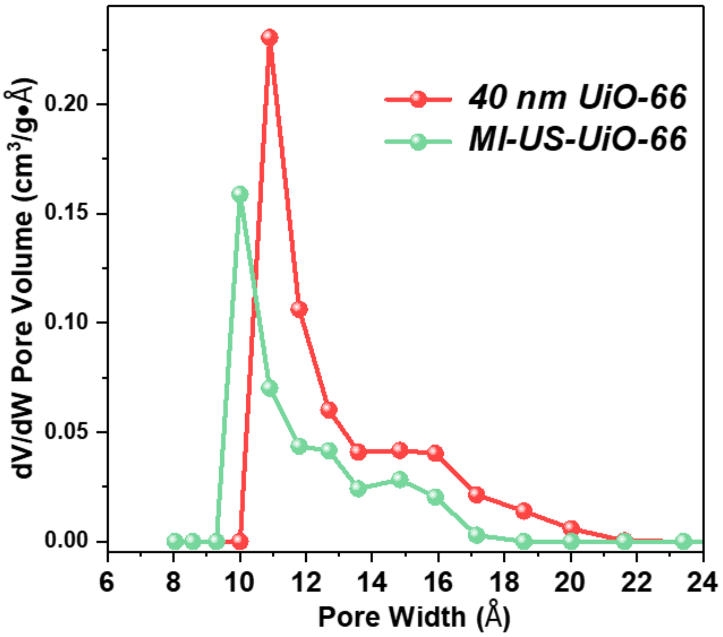

Supplementary Figure 17. Pore size distribution comparison between 40 nm UiO-66 and MI-US-UiO-66 (DFT model).

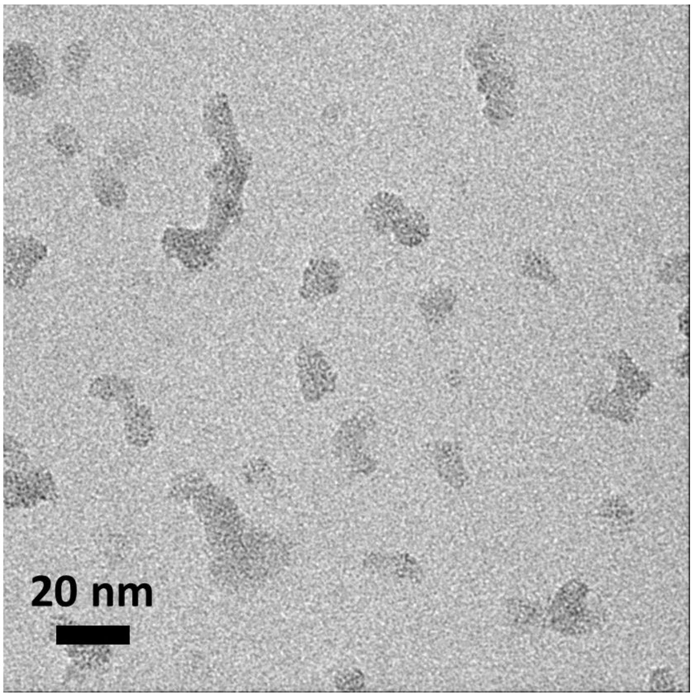

Supplementary Figure 18. TEM image of the particles synthesized using 50% DMF and 50% ethanol as solvent.

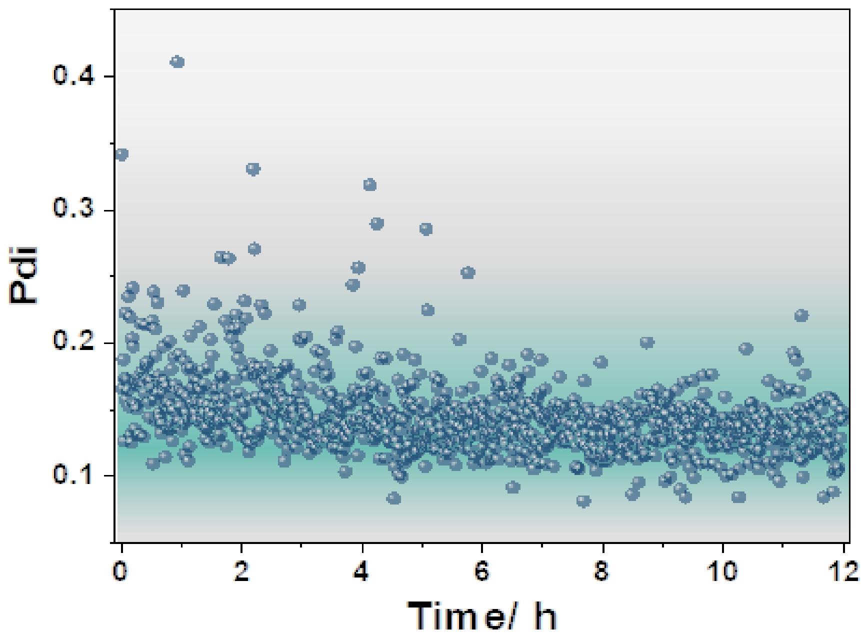

Supplementary Figure 19. Polydispersity index (Pdi) of the TD-DLS of HD-US-UiO-66 colloids (T= 25 °C).

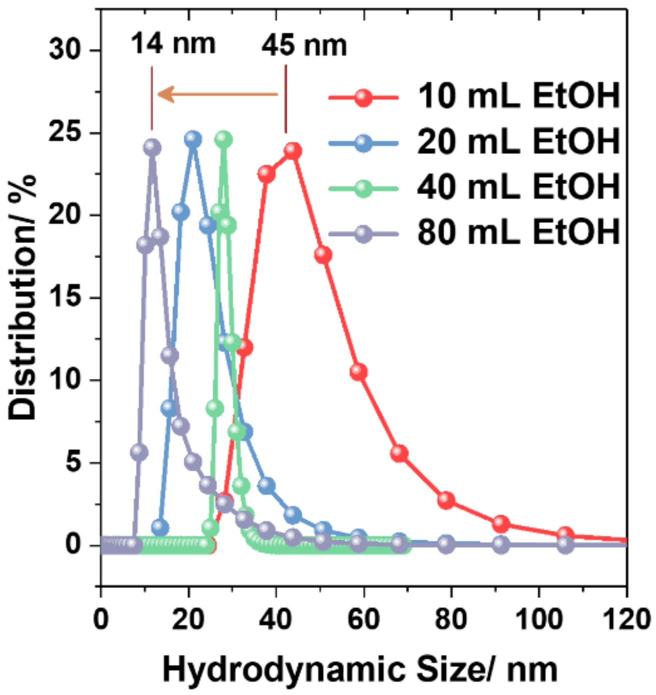

Supplementary Figure 20. The size distribution of different sized UiO-66 in water (concentration: 1 mg/ mL).

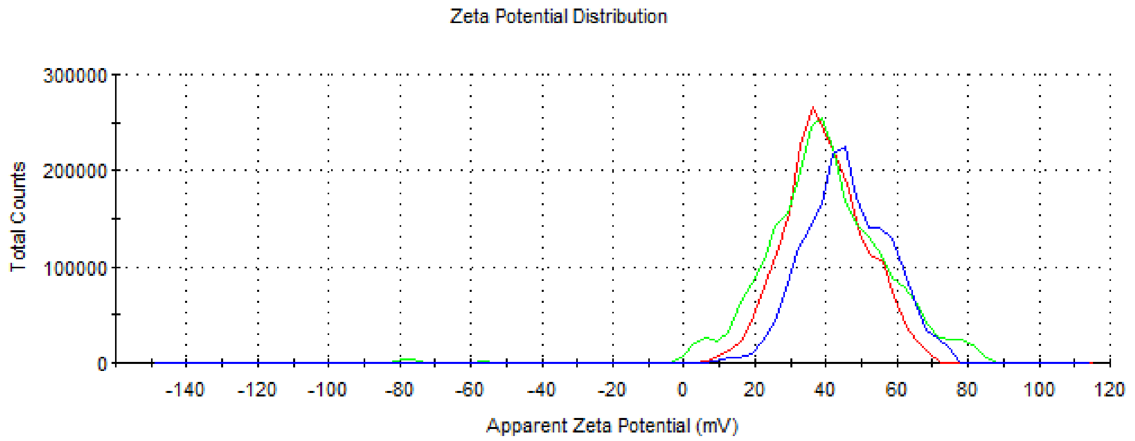

Supplementary Figure 21. Zeta potential measurements of HD-US-UiO-66 (three parallel scans).

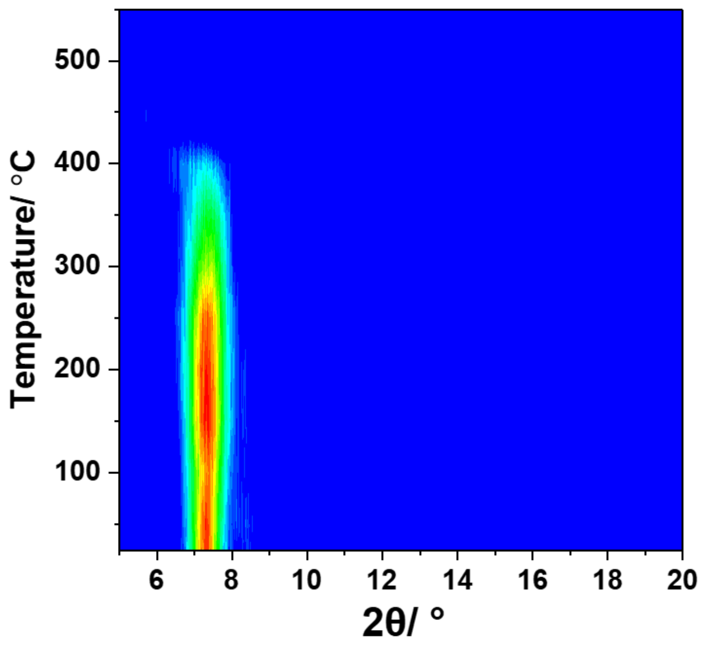

Supplementary Figure 22. In situ variable temperature PXRD patterns (λ = 1.5418 Å) of highly defective 7 nm UiO-66.

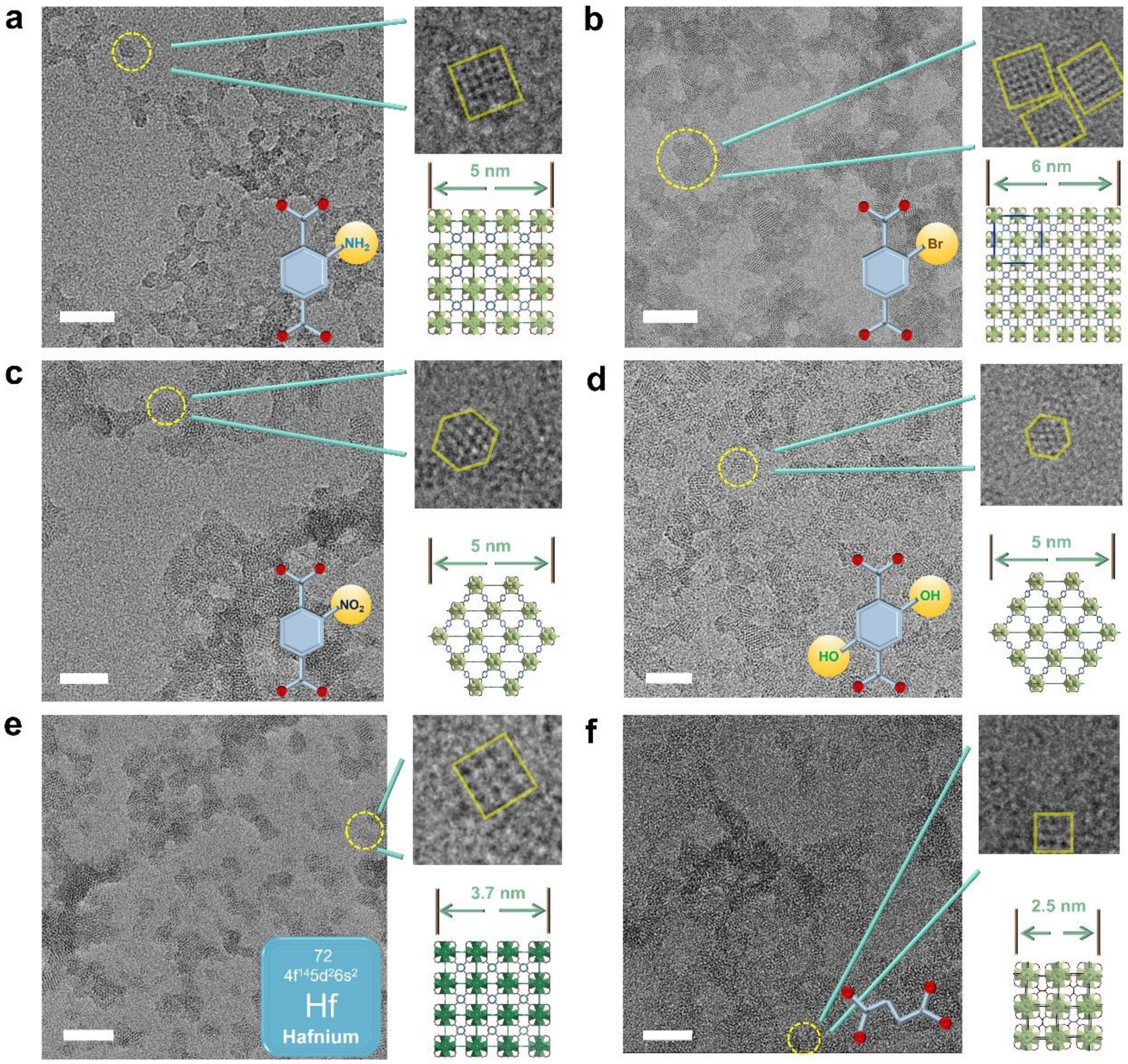

Supplementary Figure 23. Highly defective ultra-small MOFs synthesized. Low dose HRTEM images of a) HD-US-UiO-66-NH_2, b) HD-US-UiO-66-Br, c) HD-US-UiO-66-NO_2, d) HD-US-UiO-66-$(OH)_2$, e) HD-US-UiO-66(Hf), f) HD-US-MOF-801, and the zoomed-in images of individual crystals and the corresponding schematic diagram of the structures. Scale bar: 20 nm.

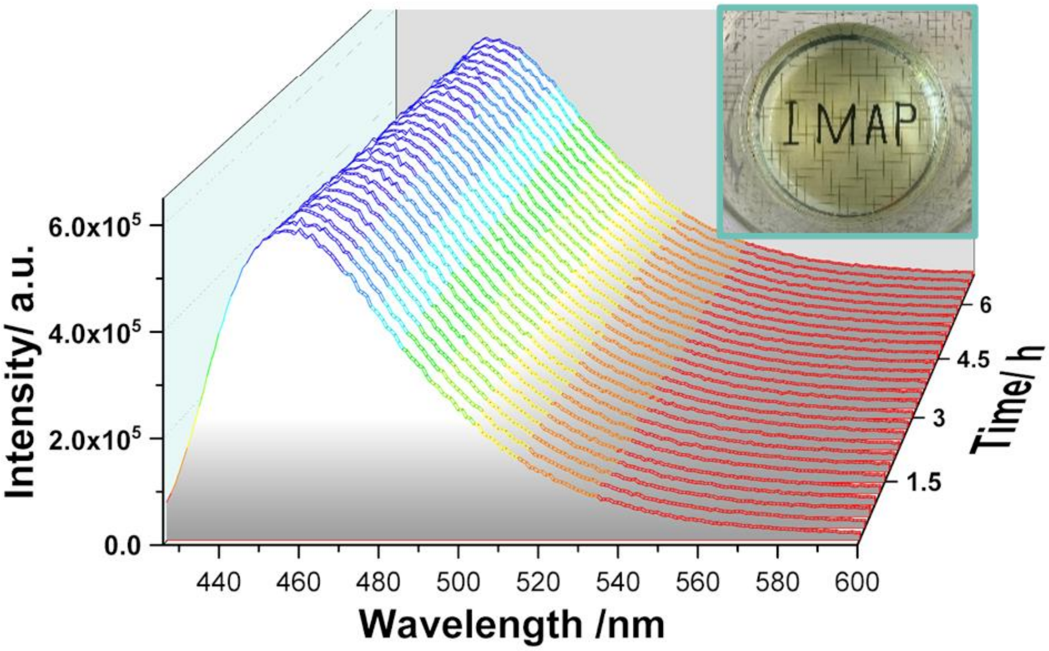

Supplementary Figure 24. Time-dependent fluorescence spectra of HD-US-UiO-66-NH_2 in water, (λ=360 nm, 5mg/mL) the insert image: transparency of the MOF, photography of a beaker with aqueous 5mg/mL HD-US-UiO-66-NH_2 on the paper with "IMAP" handwriting.

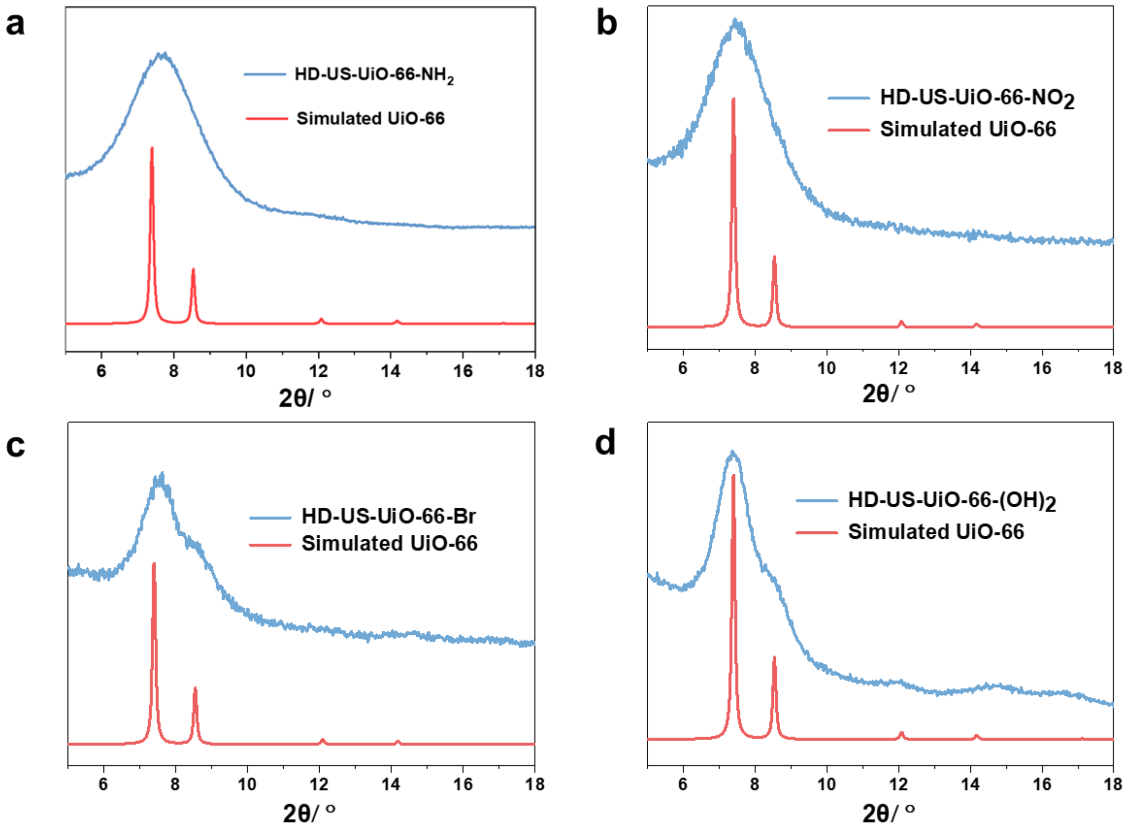

Supplementary Figure 25. PXRD patterns (λ = 1.5418 Å) of a) HD-US-UiO-66-NH_2, b) HD-US-UiO-66-NO_2, c) HD-US-UiO-66-Br, d) HD-US-UiO-66-$(OH)_2$, compared to the calculated pattern of UiO-66.

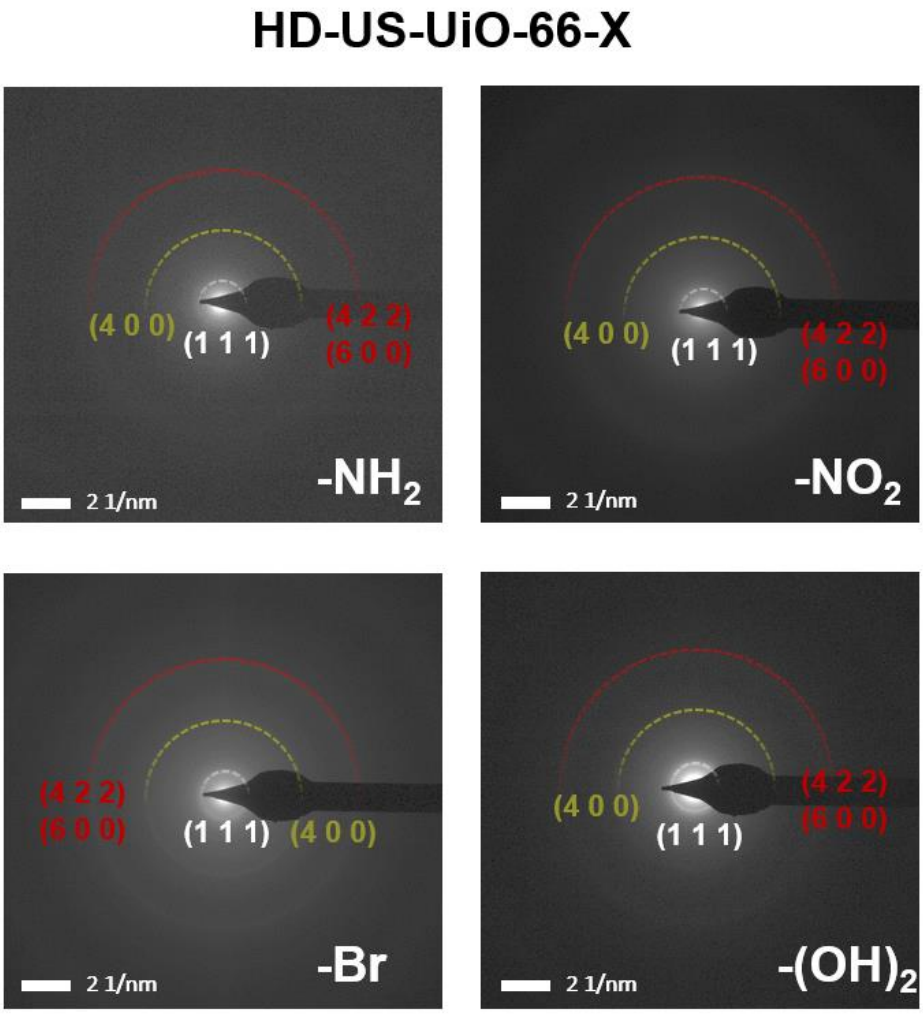

Supplementary Figure 26. SAED pattern of the 5 nm HD-US-UiO-66-X (X=NH_2, NO_2, $(OH)_2$, Br).

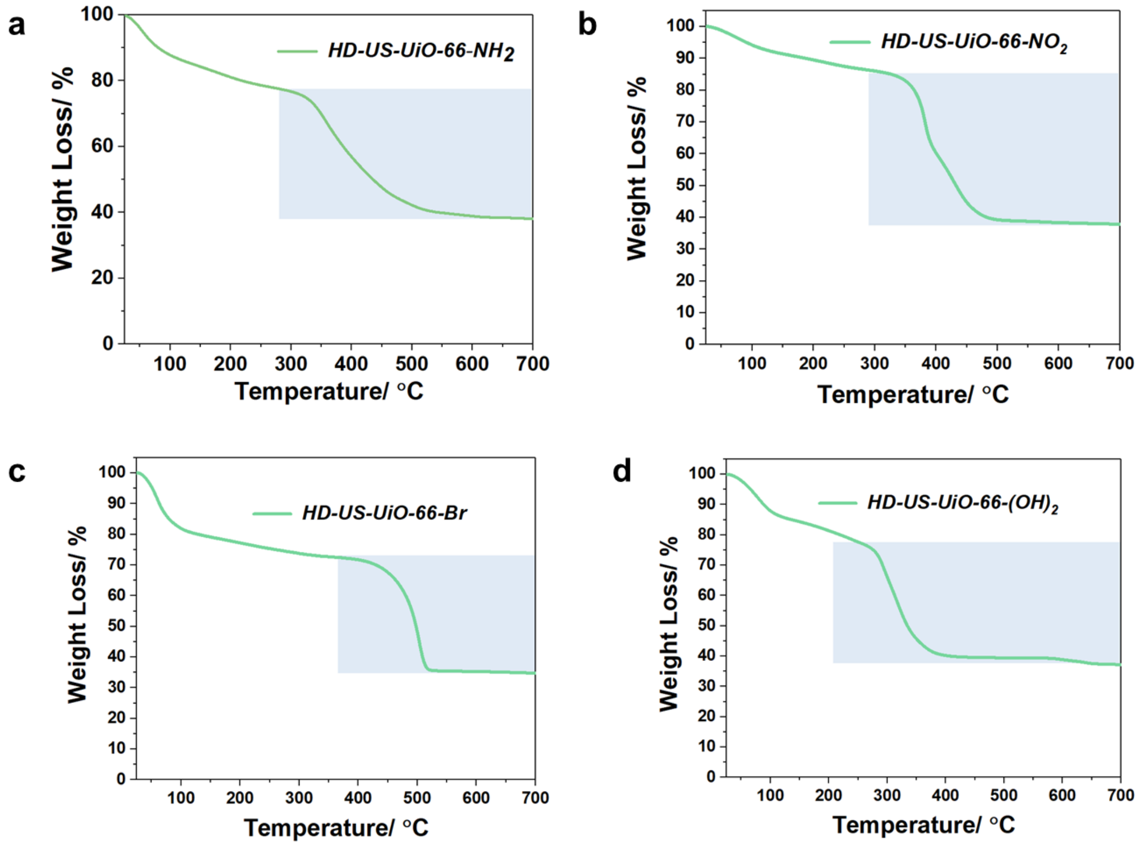

Supplementary Figure 27. TGA of a) HD-US-UiO-66-NH_2, b) HD-US-UiO-66-NO_2, c) HD-US-UiO-66-Br, d) HD-US-UiO-66-$(OH)_2$ under oxygen flow, 70 mL/min, 5 °C/min.

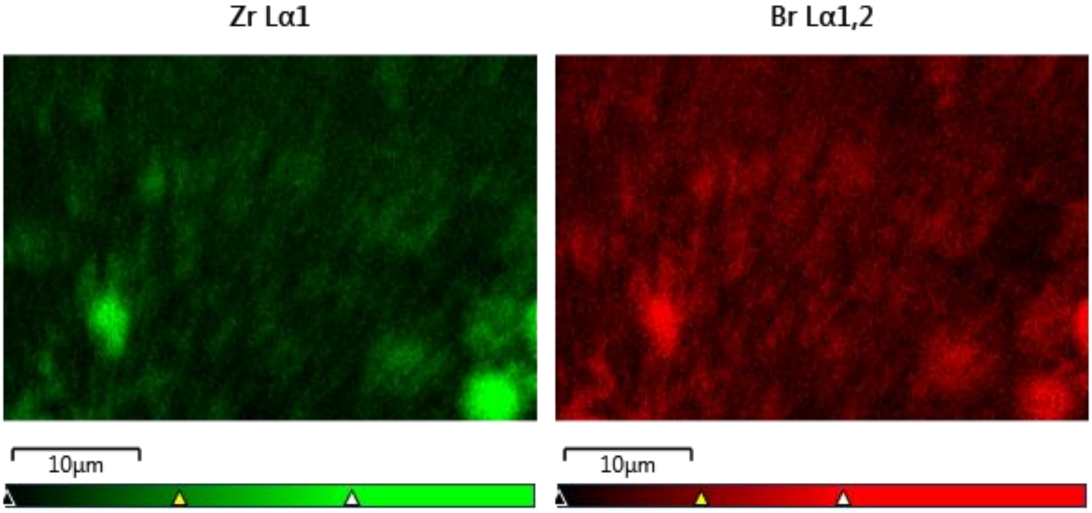

Element	Wt%	Wt% Sigma
Br	31.37	0.49
Zr	62.65	0.53

Supplementary Figure 28. EDX global mapping of HD-US-UiO-66-Br and the calculated content of Zr and Br.

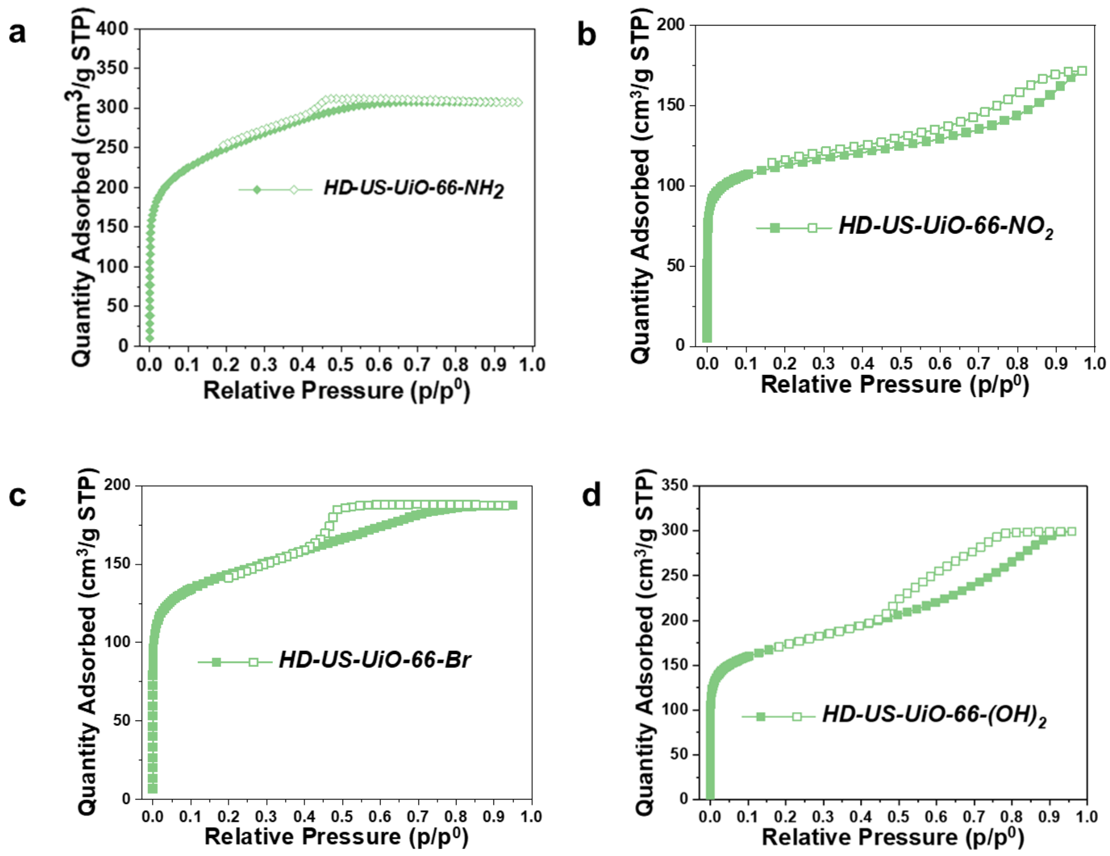

Supplementary Figure 29. 77K N_2 sorption isotherm of a) HD-US-UiO-66-NH_2, b) HD-US-UiO-66-NO_2, c) HD-US-UiO-66-Br, d) HD-US-UiO-66-$(OH)_2$, filled square for adsorption and empty square for desorption.

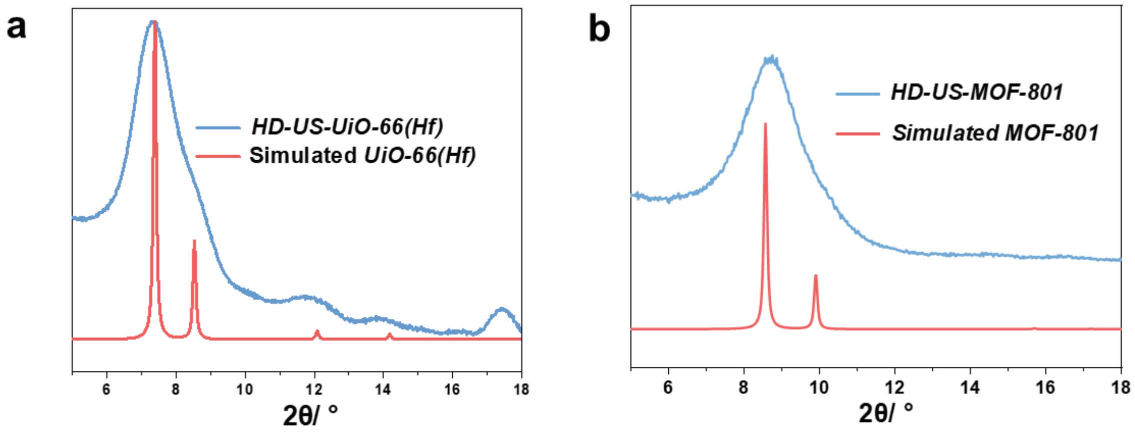

Supplementary Figure 30. PXRD patterns (λ = 1.5418 Å) of HD-US-UiO-66(Hf) and HD-US-MOF-801 compared to the corresponding calculated patterns from structures.

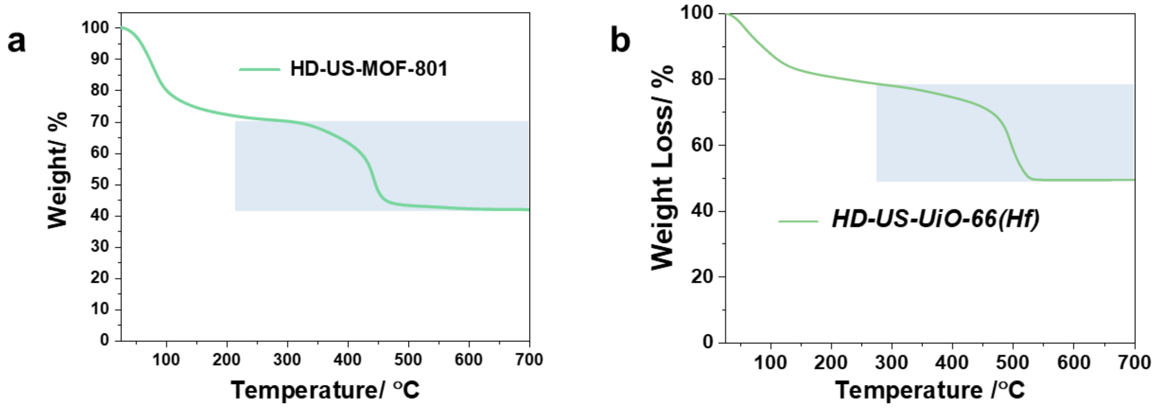

Supplementary Figure 31. TGA of HD-US-MOF-801 and HD-US-UiO-66(Hf) under oxygen flow, 70 mL/min, 5 °C/min.

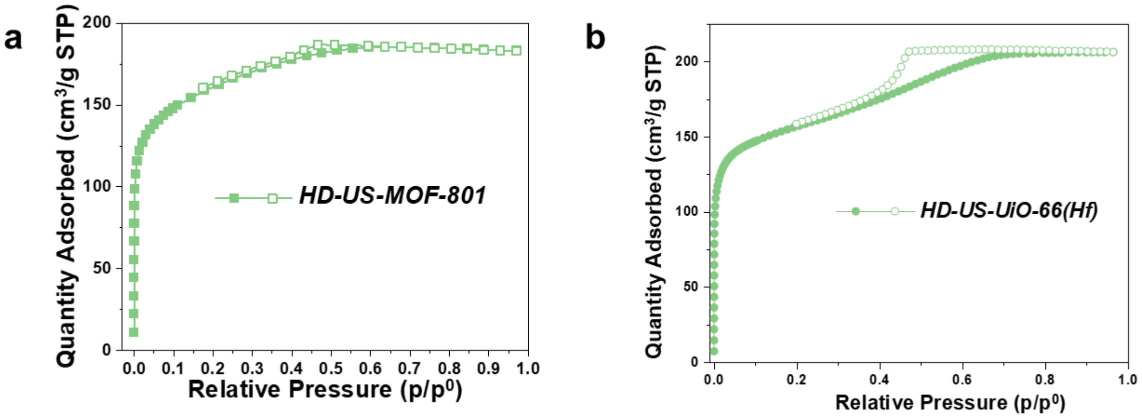

Supplementary Figure 32. 77K N_2 sorption isotherm of a) HD-US-MOF-801, b) HD-US-UiO-66(Hf), filled square for adsorption and empty square for desorption.

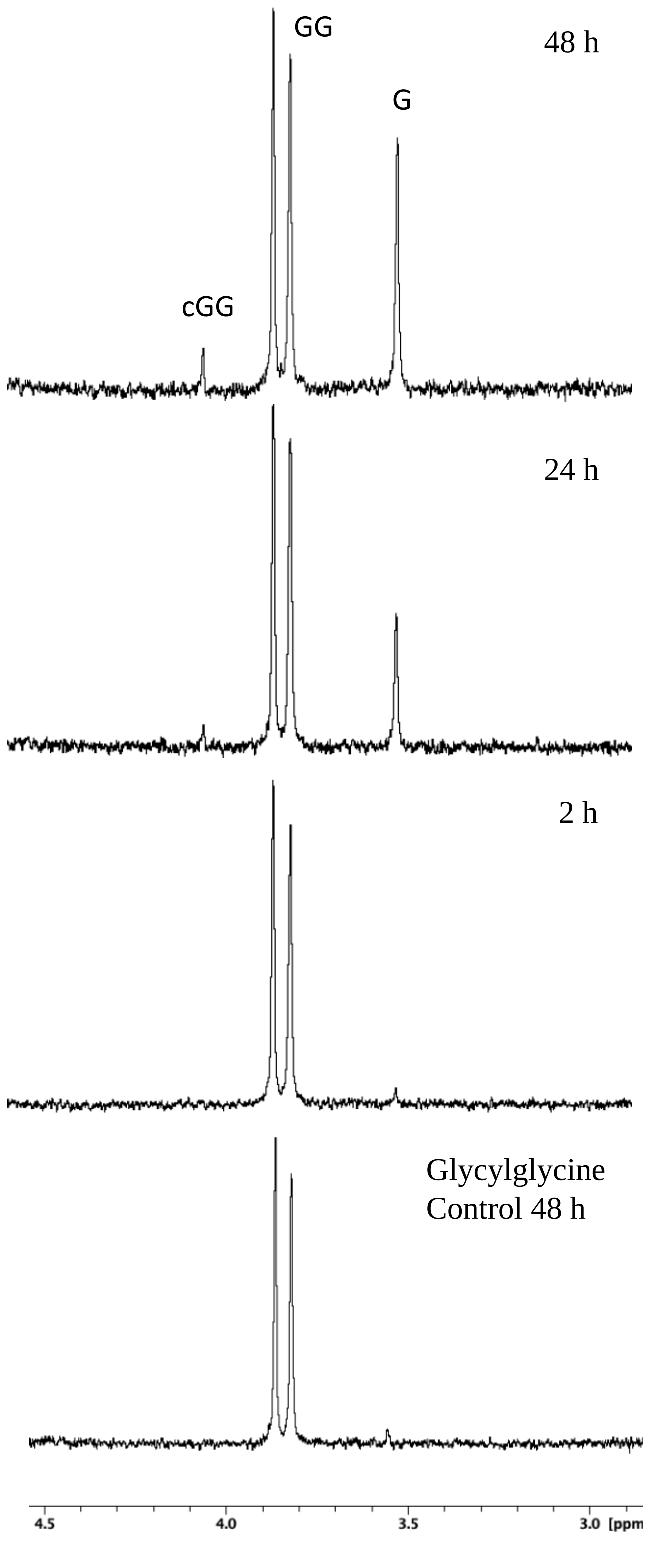

Supplementary Figure 33. ^{1}H NMR of 2 mM glycylglycine (GG) hydrolysed to glycine (G) and cyclic glycylglycine (cGG) over time in the presence of HD-US-UiO-66 D_2O, pD = 7.4, 60 °C

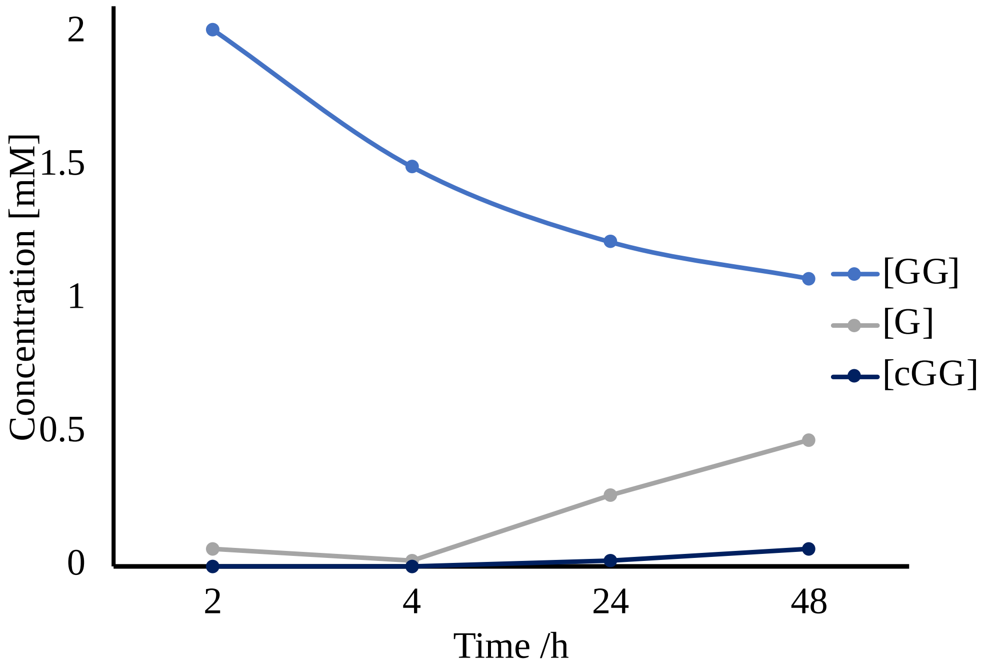

Supplementary Figure 34. Change in concentration of GG, G and cGG over time in the presence of HD-US-UiO-66, D_2O, pD = 7.4, 60 °C.

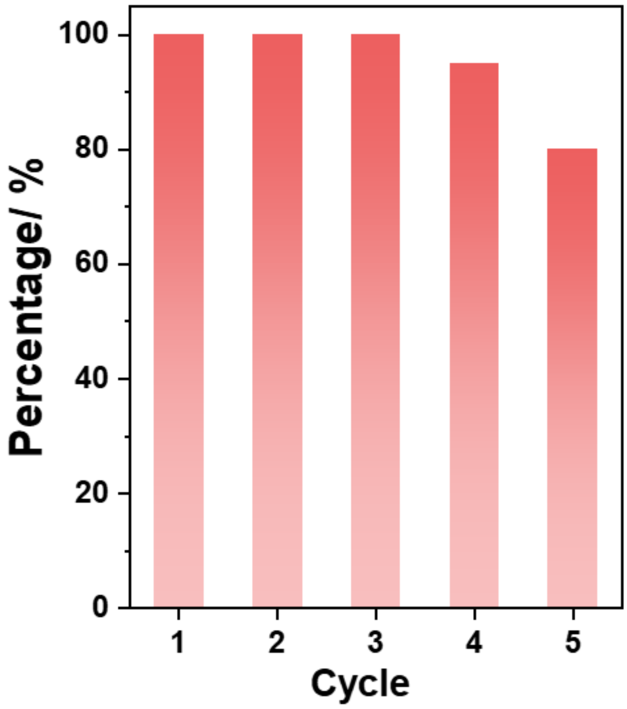

Supplementary Figure 35. Activity of HD-US-UiO-66-NH_2 towards GlyGly hydrolysis for 5 reaction cycles, percentage taken compared to rate of cycle 1.

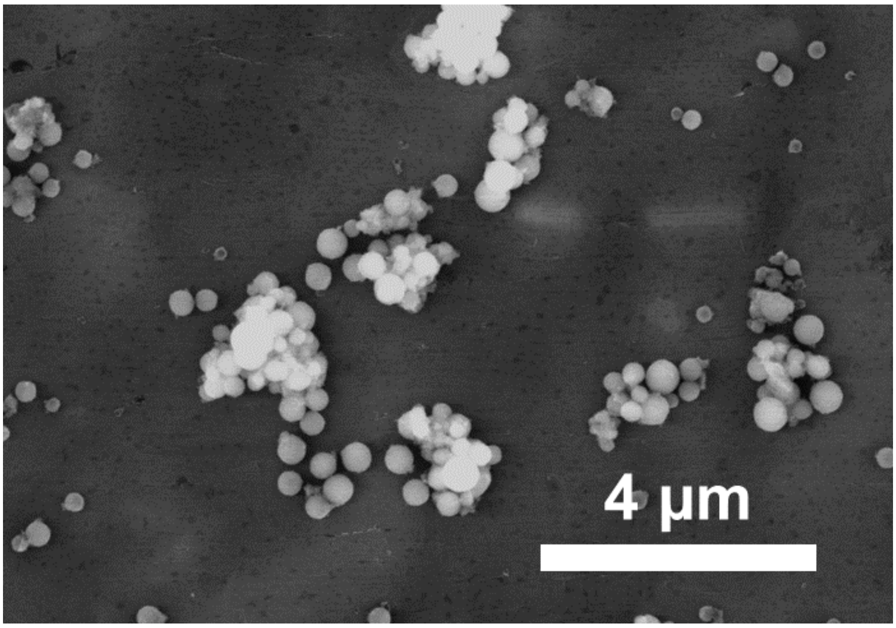

Supplementary Figure 36. SEM image of HD-200-UiO-66-NH_2.

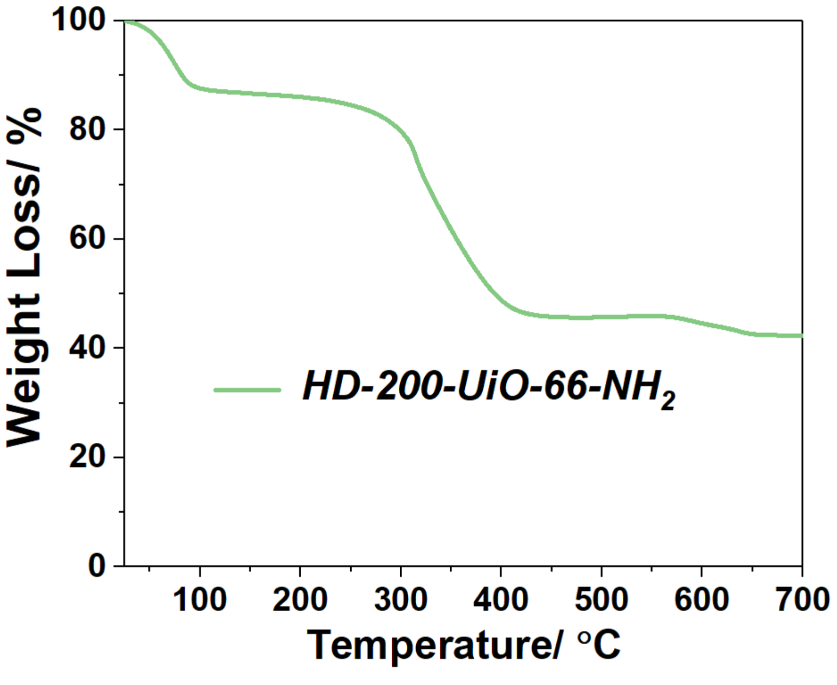

Supplementary Figure 37. TGA of HD-200-UiO-66-NH_2 under oxygen flow, 70 mL/min, 5 °C/min.

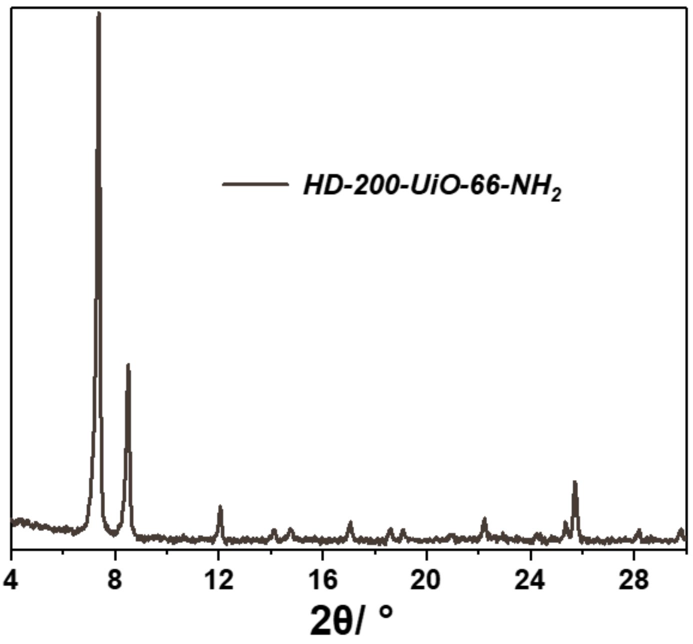

Supplementary Figure 38. PXRD pattern (λ = 1.5418 Å) of HD-200-UiO-66-NH_2.

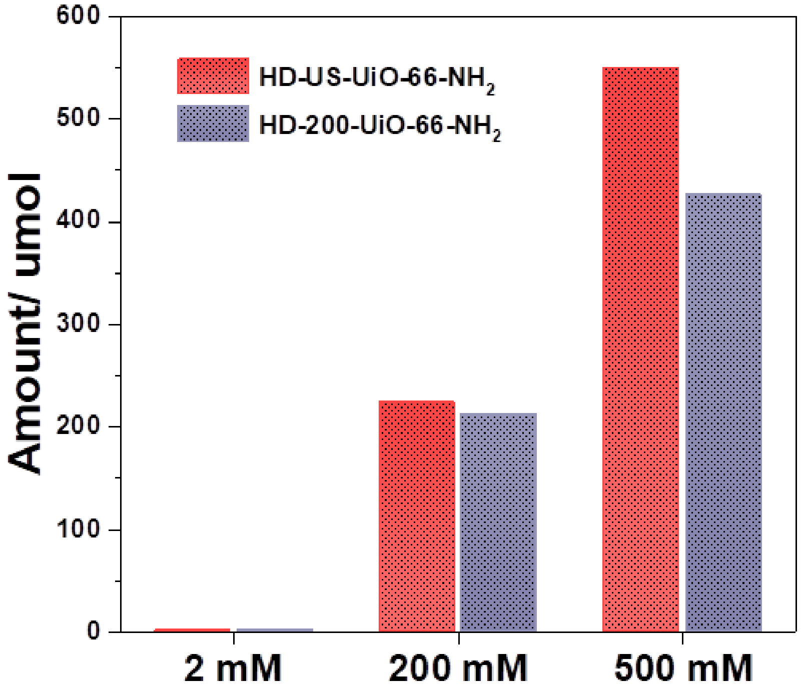

Supplementary Figure 39. Total recovery of the GG and G from different batches of GG concentration with HD-US-UiO-66-NH_2 and HD-200-UiO-66-NH_2.

Supplementary References

1. Dai S, Simms C, Dovgaliuk I, Patriarche G, Tissot A, Parac-Vogt TN, *et al.* Monodispersed MOF-808 Nanocrystals Synthesized via a Scalable Room-Temperature Approach for Efficient Heterogeneous Peptide Bond Hydrolysis. *Chem. Mater.* **33**, 7057-7066 (2021).

2. Ly HGT, Fu G, de Azambuja F, De Vos D, Parac-Vogt TN. Nanozymatic Activity of UiO-66 Metal–Organic Frameworks: Tuning the Nanopore Environment Enhances Hydrolytic Activity toward Peptide Bonds. *ACS Appl. Nano Mater.* **3**, 8931-8938 (2020).

3. Øien S, Wragg D, Reinsch H, Svelle S, Bordiga S, Lamberti C, *et al.* Detailed structure analysis of atomic positions and defects in zirconium metal–organic frameworks. *Cryst. Growth Des.* **14**, 5370-5372 (2014).

4. Vermoortele F, Bueken B, Le Bars G, Van de Voorde B, Vandichel M, Houthoofd K, *et al.* Synthesis modulation as a tool to increase the catalytic activity of metal–organic frameworks: the unique case of UiO-66 (Zr). *J. Am. Chem. Soc.* **135**,11465-11468 (2013).

5. Gutov OV, Hevia MG, Escudero-Adan EC, Shafir A. Metal–organic framework (MOF) defects under control: insights into the missing linker sites and their implication in the reactivity of zirconium-based frameworks. *Inorg. Chem.*, **54**, 8396-8400 (2015).

6. Wei R, Gaggioli CA, Li G, Islamoglu T, Zhang Z, Yu P, *et al.* Tuning the Properties of Zr6O8 Nodes in the Metal Organic Framework UiO-66 by Selection of Node-Bound Ligands and Linkers. *Chem. Mater.* **31**,1655-1663 (2019).

7. Yang D, Ortuño MA, Bernales V, Cramer CJ, Gagliardi L, Gates BC. Structure and dynamics of Zr6O8 metal–organic framework node surfaces probed with ethanol dehydration as a catalytic test reaction. *J. Am. Chem. Soc.* **140**, 3751-3759 (2018).

8. Butova VV, Burachevskaya OA, Ozhogin IV, Borodkin GS, Starikov AG, Bordiga S, *et al.* UiO-66 type MOFs with mixed-linkers-1, 4-Benzenedicarboxylate and 1, 4-naphthalenedicarboxylate: Effect of the modulator and post-synthetic exchange. *Microporous Mesoporous Mater.* **305:** 110324 (2020).

9. Liang W, Coghlan CJ, Ragon F, Rubio-Martinez M, D'Alessandro DM, Babarao R. Defect engineering of UiO-66 for CO 2 and H 2 O uptake–a combined experimental and simulation study. *Dalton Trans.* 2016, **45**,4496-4500 (2016).

10. Schaate A, Roy P, Godt A, Lippke J, Waltz F, Wiebcke M, *et al.* Modulated synthesis of Zr-based metal–organic frameworks: from nano to single crystals. *Chem. Eur. J.* **17**,6643-6651 (2011).

11. Kreno LE, Leong K, Farha OK, Allendorf M, Van Duyne RP, Hupp JT. Metal–organic framework materials as chemical sensors. *Chem. Rev.* **112**,1105-1125 (2012).
